# Determination of ferroelectric contributions to electromechanical response by frequency dependent piezoresponse force microscopy

**DOI:** 10.1038/srep30579

**Published:** 2016-07-28

**Authors:** Daehee Seol, Seongjae Park, Olexandr V. Varenyk, Shinbuhm Lee, Ho Nyung Lee, Anna N. Morozovska, Yunseok Kim

**Affiliations:** 1 School of Advanced Materials Science and Engineering, Sungkyunkwan University (SKKU), Suwon, 440-746, Republic of Korea; 2 Institute of Physics, National Academy of Sciences of Ukraine, 46, pr. Nauki, 03028 Kyiv, Ukraine; 3 Materials Science and Technology Division, Oak Ridge National Laboratory, Oak Ridge, Tennessee 37831, United States

## Abstract

Hysteresis loop analysis via piezoresponse force microscopy (PFM) is typically performed to probe the existence of ferroelectricity at the nanoscale. However, such an approach is rather complex in accurately determining the pure contribution of ferroelectricity to the PFM. Here, we suggest a facile method to discriminate the ferroelectric effect from the electromechanical (EM) response through the use of frequency dependent ac amplitude sweep with combination of hysteresis loops in PFM. Our combined study through experimental and theoretical approaches verifies that this method can be used as a new tool to differentiate the ferroelectric effect from the other factors that contribute to the EM response.

Ferroelectric materials exhibit spontaneous polarization, which can be switched by the application of an external electric field and further, possess piezoelectricity at the same time. Based on their ferroelectric and piezoelectric properties, the ferroelectric materials have been of great interest for a number of applications, including ferroelectric memory and piezoelectric energy harvesting devices^1^,^2^,^3^,^4^. As the demand for small and low-dimensional devices increases, numerous approaches have been conducted to investigate ferroelectric properties at the nanoscale^5^,^6^,^7^,^8^. Among these studies, nanoscale probing of ferroelectricity by measuring hysteresis loops via piezoresponse force microscopy (PFM), which detects the electromechanical (EM) response originated from the piezoelectricity, has been very successful^9^,^10^,^11^. In general, such measurements have provided results of the hysteresis loops that have been regarded as direct evidence for the existence of ferroelectricity. However, several groups recently demonstrated ferroelectric-like hysteresis loops in non-ferroelectric materials, such as TiO_2_, SrTiO_3_, yttrium-stabilized zirconia, CaCu_3_Ti_4_O_12_, and Li-ion conductors such as LiCoO_2_^12^,^13^,^14^,^15^,^16^,^17^,^18^,^19^,^20^. Even though some of these materials exhibit true ferroelectricity through the modulation of lattice by strain or are indirectly related to the ferroelectricity^4^,^16^,^21^, such hysteric behaviors from many of such materials, including LiCoO_2_, silicon and soda-lime glass, cannot be attributed to the piezoelectric and ferroelectric properties^13^,^22^,^23^. Recent studies have reported that unexpected ferroelectric-like hysteresis loops are primarily correlated with the Vegard strain which presents ionically induced surface strain originated from the Vegard rule^14^,^16^,^24^,^25^, and further, these properties can originate from electrostriction^25^ and/or the electrostatic influence of injected surface charges^16^,^26^.

In order to understand the pure ferroelectric effect from the various phenomena that contribute to the EM response, the PFM phase change and/or the shape of the hysteresis loops are primarily used to interpret the presence of ferroelectricity^25^,^26^. However, if the target materials do not exhibit a hysteresis loop opening with 180° phase change, this approach can be ineffective in differentiating ferroelectric effect. For example, a locally incomplete switching behavior have been reported even in conventional ferroelectric oxides^27^,^28^. In such cases, since the polarization is not fully switched, the PFM phase does not change and loop opening is not fully developed. Hence, an approach based on the hysteresis loop is limited to unambiguously extract the ferroelectric effect from the complex contributions to the EM response. Thus, it is urgent to suggest alternative and/or complementary methods that can address the ferroelectricity at the nanoscale.

Here, we suggest a simple, yet powerful approach to decouple the ferroelectric and Vegard effects through the use of frequency dependent ac amplitude sweep from PFM with combination of the hysteresis loops. The EM amplitude as a function of the ac amplitude, *i.e.* ac amplitude sweep, has been typically used to measure the piezoelectric coefficient of a piezo/ferroelectric material^29^,^30^. While the EM amplitude originated from piezoelectricity is independent on the frequency, Vegard strain is expected to be dependent on the frequency. Hence, frequency dependent ac amplitude sweep with combination of the hysteresis loops could readily discriminate ferroelectric effect from the Vegard effect that contributes to the EM response. We further investigate the origin of the EM amplitude beyond the piezoelectricity/ferroelectricity in PFM in order to demonstrate our approach. Our approach is examined by using Li-ion conductive glass ceramics (LICGCs), which have both ionic and piezoelectric phases, and ferroelectric Pb(Zr,Ti)O_3_ (PZT) as model systems. The frequency dependent EM amplitudes and hysteresis loops were experimentally observed in each model system by using PFM and then, the frequency dependent EM amplitudes were also theoretically simulated through the use of finite element method.

## Results and Discussion

**Figure 1:**
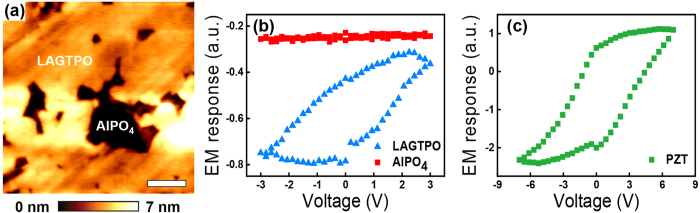
(**a**) Topography of the LICGC. (**b**,**c**) Hysteresis loops of (**b**) the AlPO_4_ and the LAGTPO in the LICGC and (**c**) the PZT thin film. Scale bar is 200 nm. Note that EM response is defined as the EM amplitude multiplied by the sine phase.

A topography of LICGC is shown in Fig. 1(a). The dark and bright regions in the image represent the AlPO_4_ and Li_x_Al_x_Ge_y_Ti_2-x-y_P_3_O_12_ (LAGTPO), respectively^31^. They are readily distinguished by their distinctly different topographical shapes, as can be seen in Fig. 1(a). Figure 1(b,c) present hysteresis loops of AlPO_4_, LAGTPO and a PZT thin film, respectively. The highly polar PZT thin film clearly shows a typical ferroelectric hysteresis loop with a large loop opening. On the other hand, there is no clear switching behavior in AlPO_4_, which is one of the most well-known characteristics of piezoelectric materials. Hence, the hysteresis loop can be used to differentiate between piezoelectric and ferroelectric materials. In the case of LAGTPO, there is a relatively large loop opening with hysteric behavior. However, the hysteresis loop was only generated on the negative EM response region. This behavior can be a result of the nonpolar nature of the Vegard strain^25^. Thus, the results obtained by conducting hysteresis loop measurements could provide some insight to differentiate the ferroelectric effect, as was previously reported elsewhere^25^. Since the mechanisms inducing the EM response in AlPO_4_, LAGTPO and a PZT thin film can be clearly distinguished by the hysteresis loop measurements and furthermore, their origins of the EM response are very well known, they are considered as suitable model systems to verify the effectiveness of our complementary approach.

**Figure 2:**
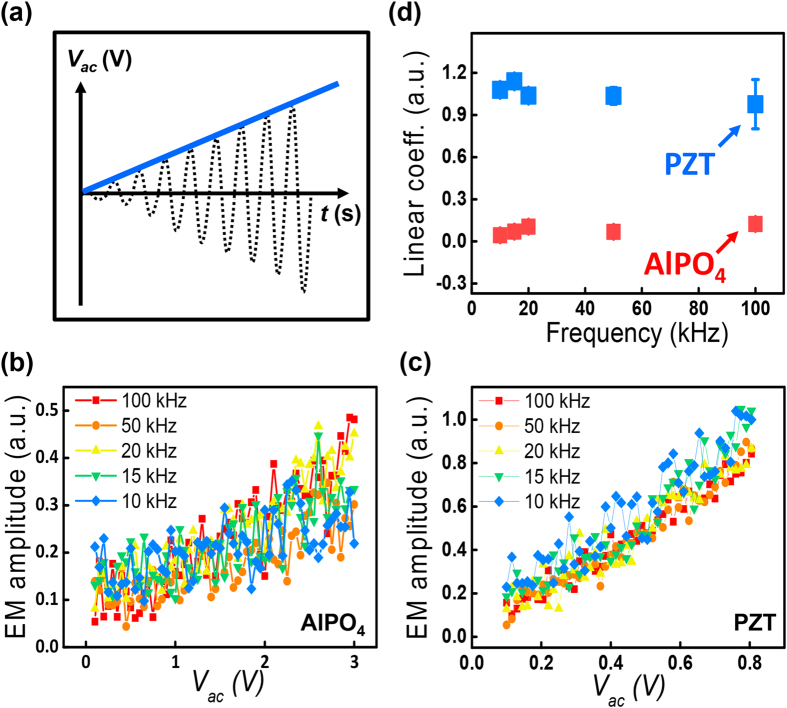
(**a**) Schematic of the the ac amplitude sweep. (**b**,**c**) EM amplitude as a function of the magnitude of the ac amplitude at various frequencies in (**b**) the AlPO_4_ of the LICGC and (**c**) the PZT thin film. The solid lines are guide lines for the eyes. (**d**) Calculated linear coefficients of the AlPO_4_ in the LICGC and the PZT thin film. Note that the EM amplitude only contains the amplitude signal.

To differentiate the ferroelectric effect from the Vegard effect that contributes to the EM response, we measured the EM amplitude at different frequencies based on the ac amplitude sweep method. Figure 2(a) shows a schematic of the ac amplitude sweep to induce the EM amplitude, which has been typically used to measure the piezoelectric coefficient of a piezo/ferroelectric material^28^,^29^. The EM amplitude obtained for the AlPO_4_ gradually increases as the magnitude of the ac amplitude increases, regardless of its frequency, as shown in Fig. 2(b). Since the EM amplitude of the AlPO_4_ showed a linear relation with the magnitude of the ac amplitude, which is a typical characteristic of piezoelectric materials, the origin of the EM amplitude in the AlPO_4_ is expected to be a piezoresponse one. In fact, as mentioned above, AlPO_4_ is piezoelectric^32^. In the case of the piezoresponse, the obtained results demonstrate that there is no distinct tendency on the frequency dependence. However, since the magnitude of the EM amplitude in the AlPO_4_ is relatively small, resulting from its low piezoelectric coefficient^32^, it is not sufficient in showing the frequency independence of the piezoresponse.

To further examine the frequency independent piezoresponse, we have selected a PZT thin film, which is a well-known ferroelectric material that has a much higher piezoelectric coefficient than AlPO_4_, as an additional model system. We note that the maximum ac voltage for the measurements was below the coercive voltage, and the ac amplitude sweep was performed on the fully upward polarized region of the as-grown sample (see Fig. S1). As shown in Fig. 2(c), there is no significant frequency dependence in the PZT thin film as well. The linear coefficients that were extracted from the linear fits of both the AlPO_4_ and the PZT thin film, *i.e.*, the piezoelectric coefficients, are shown in Fig. 2(d). In this case, the linear coefficient of the AlPO_4_ obtained at 100 kHz, 0.97 a.u. is directly related with the piezoelectric coefficient of the AlPO_4_, 3.30 pm/V^32^. This result indicates that the linear coefficients of the AlPO_4_ and the PZT thin films are almost constant over the all frequency range we have tested. It is worth mentioning that the magnitude of the EM amplitude can be changed by the involvement of the cantilever dynamics to the observed signal^33^,^34^. For instance, if the ac amplitude sweep is performed in the vicinity of the contact resonance frequency of the tip-sample junction, it can affect to the frequency dependence of the EM amplitude even though the piezoelectricity is not dependent on the frequency^34^. Thus, in this work, most of the measurements were performed under 50 kHz because this regime is far from the contact resonance frequency, which is around ~320 kHz (see method section), and hence, frequency dependence caused by the cantilever dynamics can be minimized. As a consequence, the EM amplitude observed in the AlPO_4_ and the PZT thin film, which was induced by the piezoresponse, did not exhibit any significant frequency dependence in the measured frequency range. Moreover, these results are well consistent with the previous reports^35^,^36^. We note that, since contact resonance frequency depends on the stiffness of the cantilever, frequency insensitive regime can be changed by choosing cantilevers with different stiffness. In addition, even though we focus on the out-of-plane EM amplitude, the in-plane EM amplitude is often used to examine in-plane polarization properties in the ferroelectric materials^37^,^38^. Likewise to the out-of-plane EM amplitude, the in-plane EM amplitude can be also affected by the contact resonance frequency. Further, it can be affected by additional measurement and/or instrumental issues such as a slip motion of the cantilever^35^,^36^ and the limited bandwidth of the lateral photo-detector integrated in atomic force microscopy (AFM) system^39^. Therefore, if one can choose the frequency regime which is not affected by above possible reasons, in principle, the similar approach can be applicable even for the in-plane EM amplitude.

**Figure 3:**
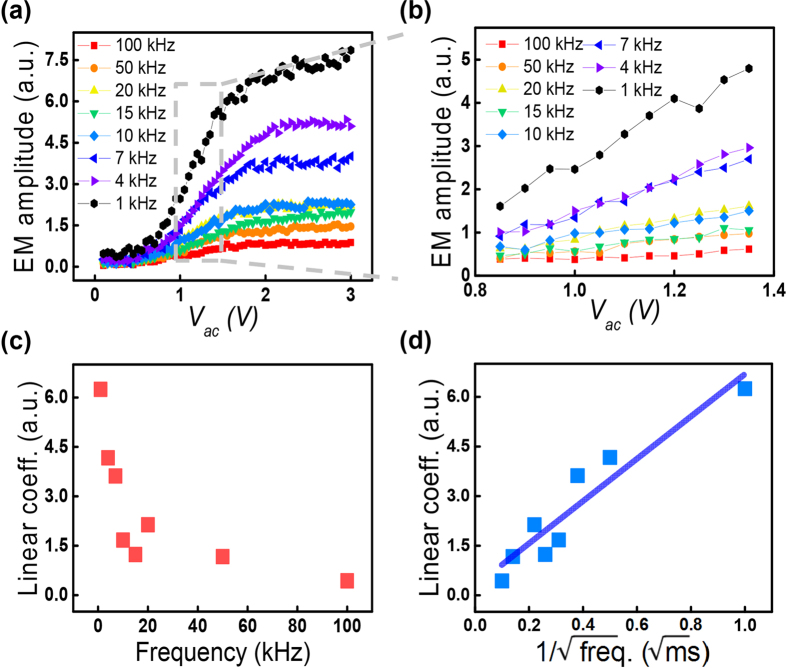
(**a**) EM amplitude of the LAGTPO of the LICGC as a function of the ac amplitude. (**b**) Linear part (gray dotted area) selected from (**a**). The solid lines are guidelines for the eyes. (**c**) Linear coefficients calculated from (**b**). (**d**) Plot of the linear coefficients as a function of the reciprocal of the square root of the frequency.

In the case of the LAGTPO, of which EM amplitude is expected to be based on the Vegard strain, the EM amplitude can show a totally different behavior compared to those of the AlPO_4_ and the PZT thin film, as shown in Fig. 3(a). At a fixed frequency of 1 kHz, the EM amplitude those was obtained slightly increases as the magnitude of the ac amplitude increases under a relatively lower ac amplitude regime, and then it rapidly increases when the magnitude of the ac amplitude becomes larger than a certain value. Finally, it shows a saturation behavior (see Fig. S3). The rapid increase in the second step can originate from the increase in the ionic movement that is induced by a higher electric field^40^, and the saturation behavior can be explained by limit to the concentration of Li ions that gather around the LAGTPO area underneath the tip and/or by the on-set of the irreversible reaction^12^,^24^,^41^. At a fixed magnitude of the ac amplitude, the EM amplitude increases as the frequency of the ac amplitude decreases over the entire range of the ac amplitude. The linear parts of the EM amplitude [gray dotted area in Fig. 3(a)] are extracted to quantify the surface displacement per unit of applied ac voltage, and these are compared to those obtained from Fig. 2(d), as described in Fig. 3(b). Figure 3(c) shows that the linear coefficients of the EM amplitude, *i.e.* electrochemical strain coefficients^42^, increase significantly as the frequency decreases. In other words, there is strong frequency dependence. Since it is well known that the Vegard response, which is induced by the movement of ions, increases as the frequency decreases, the tendency obtained for the LAGTPO can be well understood^24^.

To further explore the relationship between the frequency and the linear coefficients in the higher frequency range, the equation for the surface displacement induced by ionic motion is employed as follows:^13^


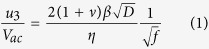


where *u*_*3*_, *ν*, *β*, *D*, *η* and *f* are the amplitude of the surface displacement, Poisson’s ratio, effective Vegard coefficient, the diffusion coefficient of Li ions, linear relation between chemical potential and applied electric field and frequency, respectively. According to the above equation, the surface displacement per unit of applied voltage is proportional to the reciprocal of the square root of the frequency, so a set of linear coefficients can be plotted as shown in Fig. 3(d). This figure clearly shows a linear relation between the surface strain per unit voltage and the frequency, underpinning the theoretical Equation (1). The slope of the fitted line as shown in Fig. 3(d) can be calibrated as 45.15 pm/V∙ms^1/2^, corresponding to *2(1 + ν)βD*^*1/2*^*/η*, through a calibration procedure based on the force-distance curve to quantitatively examine the relation between the experimental results and the theoretical equation^43^. In this case, by roughly assuming *ν* ∼ 0.3, *β* ∼ 0.05 and *D* ∼ 10^−16^ m^2^/s^24^, *η* can be estimated as a 0.91 V. This value can provide empirical insight into the relation between chemical potential and applied electric field^13^.

**Figure 4:**
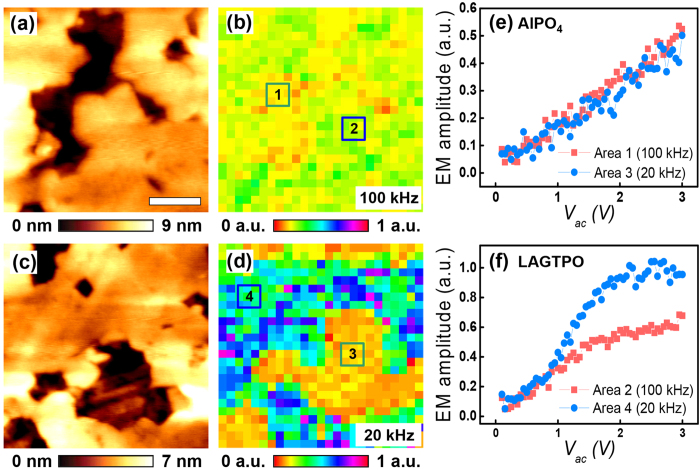
(**a**,**c**) Topography images of the LICGCs. (**b**,**d**) Spatial maps of the linear coefficient at (**b**) 100 and (**d**) 20 kHz, which correspond to (**a,c**). (**e**,**f**) Corresponding local EM amplitude as a function of the ac amplitude at each area of the (**e**) AlPO_4_ and (**f**) LAGTPO phases. Scale bar is 160 nm.

Since the LICGCs have both ionic (LAGTPO) and piezoelectric (AlPO_4_) phases, we further attempted to obtain spatially resolved frequency dependent EM amplitude information at the surface of the LICGC for directly visualizing different dependence at the ionic and piezoelectric phases. To acquire the spatial maps, the image of the topography shown in Fig. 4(a,c) was divided into a 25 by 25 square grid, and then, an ac waveform was applied at each grid point. The obtained spatial maps of the linear coefficient are shown in Fig. 4(b,d). As can be seen in Fig. 4(b,d), the two different phases of the LAGTPO and the AlPO_4_ are clearly distinguished in the spatial maps. In particular, the spatial map at lower frequency (Fig. 4(d)) shows more distinct difference between two phases because of much higher EM amplitude in the LAGTPO. Furthermore, the local EM amplitude as a function of the ac amplitude in Fig. 4(e,f) also shows different EM amplitude for each phase. Overall, unlike the piezoresponse, the EM amplitude induced by Vegard effect in the LAGTPO showed an obvious dependence on frequency. Consequently, since piezoresponse and Vegard strain, which can be considered as dominant contributions to the EM amplitude, show significantly different behavior according to the frequency, the frequency dependent ac amplitude sweep with combination of the hysteresis loops can be used to differentiate the ferroelectric effect from Vegard effect to the EM amplitude.

**Figure 5:**
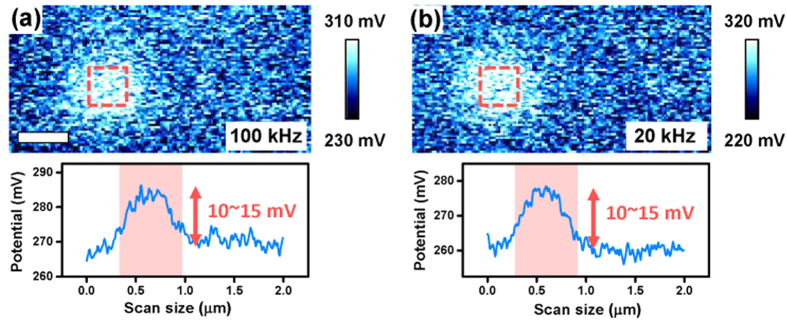
Surface potential images and corresponding average line profiles after the ac amplitude sweep. The ac amplitude sweep were performed on the red dotted boxes with 6 by 6 grid squares at (**a**) 100 kHz (**b**) 20 kHz over areas of 300 nm by 300 nm. Note that the distance between central positions of the grid squares is 50 nm which is the same with those for the measurements in Figs 2 and 3. Scale bar is 330 nm.

As mentioned in the introduction, unexpected ferroelectric-like hysteresis loops can originate from the electrostriction and/or the electrostatic influence of injected surface charges^16^,^25^,^26^. To investigate electrostatic contribution to the EM amplitude, surface potential was measured through the Kelvin probe force microscopy (KPFM) on the LAGTPO after the ac amplitude sweep. The ac amplitude sweep with 100 and 20 kHz were performed on the 6 by 6 grid squares over areas of 300 nm by 300 nm. The obtained surface potential images and corresponding average line profiles are shown in Fig. 5. The obtained results show that, after the ac amplitude sweep, the surface potential is slightly increased of around 10 ∼ 15 mV for both cases (see red dotted boxes in Fig. 5). The slightly increased surface potential is attributed to the charge injection on the sample surface through AFM tip during the ac amplitude sweep^44^. We note that the standard deviation of the surface potential image is about 7 ∼ 9 mV. This indicates that, even though there is a slight change in the surface potential, the absolute amount of the change in the surface potential is fairly small and there is no distinct difference between two different frequencies. In particular, in our case, since a relatively small ac voltage (relative to the coercive voltage) with a high frequency (relative to the dc voltage sweep frequency for the hysteresis loop) was applied to the sample, the electrostatic contribution to the EM amplitude is expected to be fairly small.

**Figure 6:**
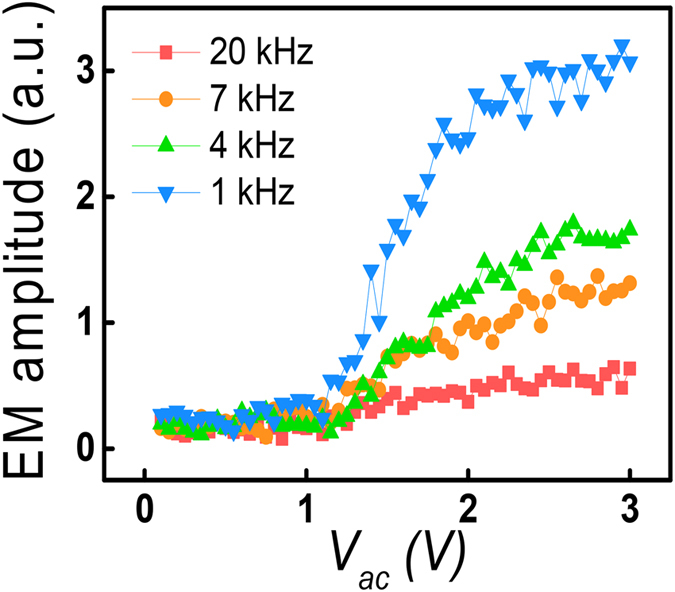
EM amplitude of the LAGTPO of the LICGC as a function of the ac amplitude obtained by soft cantilever (spring constant of ∼0.2 N/m).

Even though the absolute amount of the change in the surface potential is fairly small, actual surface potential can be larger than the observed potential if there is a strong and fast charge relaxation. Thus, to clearly confirm the influence of electrostatic contribution on the frequency dependence, we measured frequency dependence by using a soft cantilever (spring constant of ∼0.2 N/m) in the LAGTPO phases because it is expected that electrostatic contribution becomes larger in a soft cantilever^45^. The obtained results as presented in Fig. 6 exhibit fairly similar feature with that obtained using a relatively stiff cantilever (spring constant of ∼3 N/m) (Fig. 3). These results indicate that the frequency dependence of the EM amplitude is still distinguishable even using a soft cantilever. Thus, distinct features of the frequency dependent EM amplitude might not be significantly affected by the electrostatic contribution.

To further understand the frequency dependent EM amplitude, a theoretical simulation based on thermodynamics was carried out through finite element method. We first consider the analytical estimates for the 1D case of the contribution of flexoelectric, electrostrictive and Vegard response, which can be dominant mechanisms of the frequency dependent EM amplitude. We assumed that the local strain *u*_*ij*_ is composed of a summation of each mechanism:


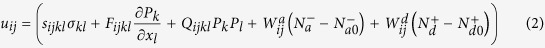


where *s*_*ijkl*_, *σ*_*kl*_, *F*_*ijki*_, *Q*_*ijki*_ and *P*_*k*_(**r**) are the elastic compliance tensor, elastic stress, flexoelectric stress tensor, electrostriction stress tensor, and polarization component, respectively. 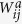 and 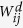 are the Vegard tensors for acceptors and donors, 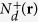 and 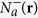 are the inhomogeneous concentrations of ionized donors and acceptors, and 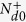 and 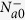 are their equilibrium concentrations. For the semiconductor film with mixed ionic-electronic conductivity, the electric potential *φ* can be found self-consistently from the Poisson equation with the short-circuited electric boundary conditions:


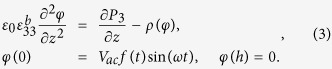


where *ε*_*0*_ and *ε* are the dielectric permittivity of vacuum and dielectric permittivity of mixed ionic-electronic conductor (MIEC), respectively. In this case, it is regarded that the film is placed in a planar capacitor under ac voltage *V*_*ac *_*f*(*t*)sin(ω*t*). In order to calculate polarization components *P*_*m*_ dependence on the electric field *E*_*m*_, we consider that the dependence using a linear dielectric (LD) approximation with the linear relation *P* = ε_0_(ε − 1)*E*_*m*_ is valid only at small applied voltages. Alternative Langeven type (LT) approximation is 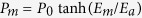 where the saturation polarization and acting field are *P*_0_ = ε_0_(ε − 1)*E*_*a*_ and *E*_*a*_, respectively. In this case, the polarization amplitude *P*_0_ and acting field *E*_*a*_ are estimated by assuming a dielectric permittivity *ε* of 5–50, screening radius *R*_*d*_ of 3 nm and applied voltage *U* of 1 V. Then, the polarization amplitude *P*_0_ and the acting field *E*_*a*_ are approximately obtained as 0.013–0.13 C/m^2^ (from *P*_0_ = ε_0_(ε − 1)*E*_*a*_) and ∼0.3 V/nm (from *E*_*a*_ ∼ *U*/*Rd*), respectively. The *dc* components, Debye (or Tomas-Fermi) approximation and *ac* components are determined by using the depletion/accumulation layer approximation. Then, the film strain and surface displacement caused by flexoelectric coupling in the LD approximation and for LT approximation are estimated. Further details can be found in the supplementary information and recent work published by Varenyk *et al*.^46^.

**Figure 7:**
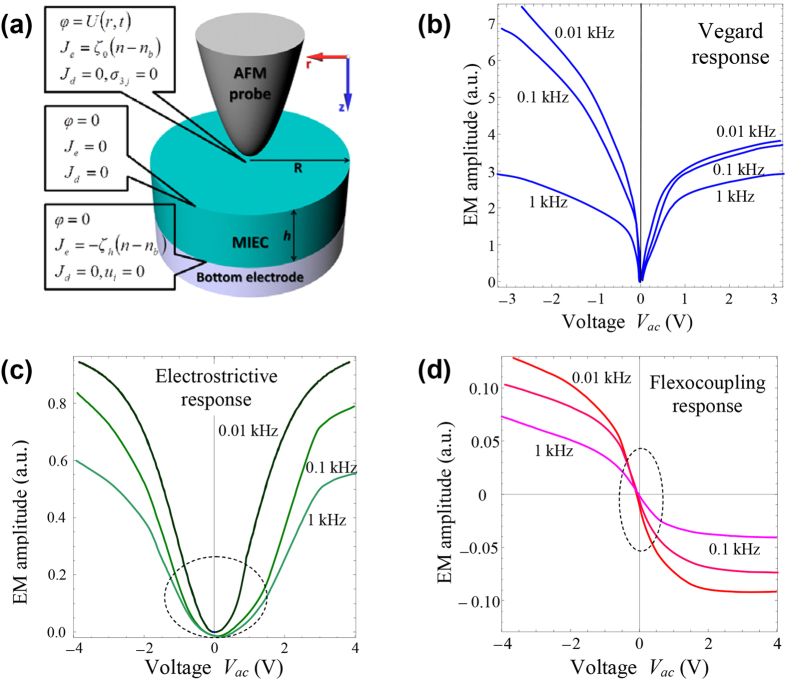
(**a**) Typical cylindrical geometry used for the simulation. The definite electrical and mechanical boundary conditions are labelled, where *φ, U, J* and *ξ* are the electric potential, the applied voltage through the tip, the current component and the positive rate constants, respectively. Note that detailed boundary conditions are provided in the supplementary information. (**b**–**d**) EM amplitudes simulated by taking into account of only (**b**) the Vegard, (**c**) electrostrictive, and (**d**) flexoelectric contributions, respectively. The different curves in (**b–d**) correspond to different frequencies of 0.01 kHz, 0.1 kHz and 1 kHz, as indicated by labels on the curves.

On the basis of these analytical estimates for the 1 D case, we consider a cylindrical problem of a nonlinear drift-diffusion kinetics model that allows for the Vegard mechanism, electrostriction, and steric limit for the mobile ion concentration and Fermi-Dirac distribution function of the electron density. Thus, the most common forms of charge species, that are nonlinearly inherent to the system, are included^47^. Finite element method was performed at various frequencies for the cylindrical geometry with the axially symmetric tip shown in Fig. 7(a). All of the physical quantities depend only on the distances z from the tip-surface interface and the polar radius r (2D problem). The redistribution of the mobile charge carriers creates an internal electric field, of which radial and normal components, 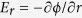 and 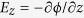, respectively, are determined from the Poisson equation for electric potential *φ*, written in the cylindrical coordinate frame as:


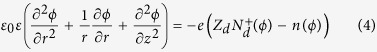


where e, n, 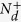 and *Z*_*d*_ are the electron charge, electron density, positively charged defect concentration and their charge in units of electron charge, respectively. The equation for the mechanical displacement can be derived from the previous analysis of continuity equations for mobile charged defects and free electrons, which are inherent to the film and the generalized Hooke’s law:


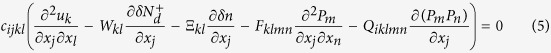


where *c*_*ijkl*_ and 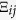 are the elastic stiffness tensor and deformation potential tensor regarded as diagonal for a numerical estimation, respectively. Variations are 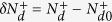 and 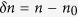. The electrical and mechanical boundary conditions assumed in theoretical calculation are provided as associated equations in Fig. 7(a). The detailed parameters used in this calculation can be found in the supplementary information.

Figure 7(b–d) show the Vegard, electrostrictive and flexoelectric contributed to the EM amplitudes, which are derived from Equation (5), when we assume that there is only one contribution. As shown in Fig. 7, the frequency dependent amplitude tends to increase as the frequency decreases regardless of the type of mechanism, which is consistent with the experimental results. Thus, it indicates that the electrostrictive and flexoelectric contributions can be further differentiated based on the frequency dependent ac amplitude sweep because these contributions are dependent on the frequency as well. Even though we consider three different contributions for the theoretical calculation, the Vegard contribution is dominant among the various contributions because its amplitude is about one to two orders of magnitude larger than the other contributions. Thus, it proves that the frequency dependent ac amplitude sweep can be used to decouple the ferroelectric and Vegard effects. We note that the frequency dependent Vegard response is very similar to the EM amplitude of the LAGTPO at the higher frequency of 100 kHz, as seen in Fig. 3(a) (see also Fig. S3 (b)). It is considered that the theoretical calculation is well consistent with experimental results. However, the EM amplitude of the LAGTPO at lower frequencies, such as 1 kHz in Figs 3(a) and S3(a), shows a somewhat different behavior in the lower voltage regimes. Even though the other contributions, such as the electrostrictive and flexoelectric response, are fairly small, they could potentially contribute to the obtained response. In addition, we note that, since the numerical simulations were performed in the nonlinear case as shown in equations A.6 (see, supplementary information), frequency dependence can be shown within kHz range. However, the electrostrictive contribution experimentally may not show the frequency dependence within kHz range. Nonetheless, in all cases, the Vegard response is expected to be primarily dominant in contributing to the frequency dependence of EM amplitude.

## Conclusion

In conclusion, we have explored the frequency dependent EM amplitudes using PFM with combination of the hysteresis loops to differentiate the ferroelectric effect from Vegard effect to the EM response. This frequency dependent approach unambiguously found that the piezoresponse in the piezoelectric and ferroelectric materials did not exhibit any significant frequency dependence whereas the Vegard strain revealed strong frequency dependence. The theoretical calculations indeed systematically confirmed the frequency dependence of the EM amplitude. Further, the theoretical calculations show that this approach can be extended to differentiate ferroelectric effect from Vegard, electrostrictive and flexoelectric contributions. This agreement clearly indicates that the frequency dependent ac amplitude sweep with combination of the hysteresis loop can be effectively used to investigate the ferroelectric contributions to the EM response, helping identify the true contributing factors to the EM response.

## Methods

### Materials

LICGCs are commercially-available solid electrolytes, which are mainly composed of Li-ionic (Li_x_Al_x_Ge_y_Ti_2−x−y_P_3_O_12_) and piezoelectric (AlPO_4_) phases and were purchased from the Ohara Corporation^48^. Since two different phases can be distinguished by their topographic shapes, this model system is suitable for our EM measurements. 50 nm-thick epitaxial PZT thin films were fabricated via pulsed laser deposition on 0.5% Nb-doped SrTiO_3_ (001) substrate (growth temperature : 625 °C, O_2_ pressure: 100 mTorr, laser repetition rate: 5 Hz). The details on the fabrication of the PZT thin film can be found elsewhere^49^.

### Measurements

The hysteresis loop and the frequency dependent ac amplitude sweep were performed using a commercial AFM (NX10, Park Systems) with a Pt coated stiff (Multi75E-G, BudgetSensors) and soft (CONTPt, Nanoworld) conductive probes of which spring constant are 3 N/m and 0.2 N/m, respectively. To measure the hysteresis loop, a function generator (PXIe-1062Q, National Instruments) controlled by the LabVIEW software was additionally equipped with the AFM to use band excitation (BE) techniques^50^. The dc voltage with a range of −7 (−3) and 7 (3) V and BE waveform of 2 (1) *V*_*pp*_ with 280–360 kHz (contact resonance frequency ∼320 kHz) were applied to the LICGCs (PZT). The hysteresis loop was averaged from a sampling of 25 measurements of different locations for each sample. In the frequency dependent ac amplitude sweep, a commercial lock-in amplifier (SR830, Stanford Research) controlled by the LabVIEW software was equipped to apply an ac bias. The ac amplitude sweep was performed as follows: The electric field was induced by a gradual increase in the ac bias, which causes periodic EM amplitude of the materials, *i.e.,* surface expansion and contraction. Then, the obtained EM amplitude is plotted as a function of the magnitude of the ac amplitude. The same measurements were performed at various frequency range and were averaged from a sampling of 4 measurements of different locations at each frequency are shown in Figs 2, 3 and 6. The ac bias of maximum 3 (0.8) V with various frequencies were applied to the LICGCs (PZT). The amplitude modulated KPFM measurements were carried out by the same AFM system. The surface potential image was obtained by a lift mode (distance: 50 nm) and the ac modulation bias 2.0 *V*_*rms*_ at 17 kHz and dc feedback bias were applied to the tip.

## Additional Information

**How to cite this article**: Seol, D. *et al*. Determination of ferroelectric contributions to electromechanical response by frequency dependent piezoresponse force microscopy. *Sci. Rep.* **6**, 30579; doi: 10.1038/srep30579 (2016).

## Supplementary Material

Supplementary Information
