# Supplementary material for: Determination of ferroelectric contributions to electromechanical response by frequency dependent piezoresponse force microscopy
Source: Sci Rep. 2016 Jul 28;6:30579. doi: 10.1038/srep30579 (PMC4964340; doi:10.1038/srep30579)
Supplement: Supplementary Information [file srep30579-s1.doc]

Supplementary Information

**Determination of ferroelectric contributions to electromechanical response by frequency dependent piezoresponse force microscopy**

*Daehee Seol,^1,+^*  *Seongjae Park,^1,+^* *Olexandr V. Varenyk,^2^ Shinbuhm Lee,^3^*

*Ho Nyung Lee,^3^ Anna N. Morozovska,^2^ and Yunseok Kim^1*^*

*^1^ School of Advanced Materials Science and Engineering, Sungkyunkwan University (SKKU), Suwon, 440-746, Republic of Korea.*

*^2^ Institute of Physics, National Academy of Sciences of Ukraine, 46, pr. Nauki, 03028 Kyiv, Ukraine.*

*^3^ Materials Science and Technology Division, Oak Ridge National Laboratory, Oak Ridge, Tennessee 37831, United States.*

+ *D. S. and S. P. contributed equally to this work.*

*** Address correspondence to *yunseokkim@skku.edu*

**I. Experimental details**


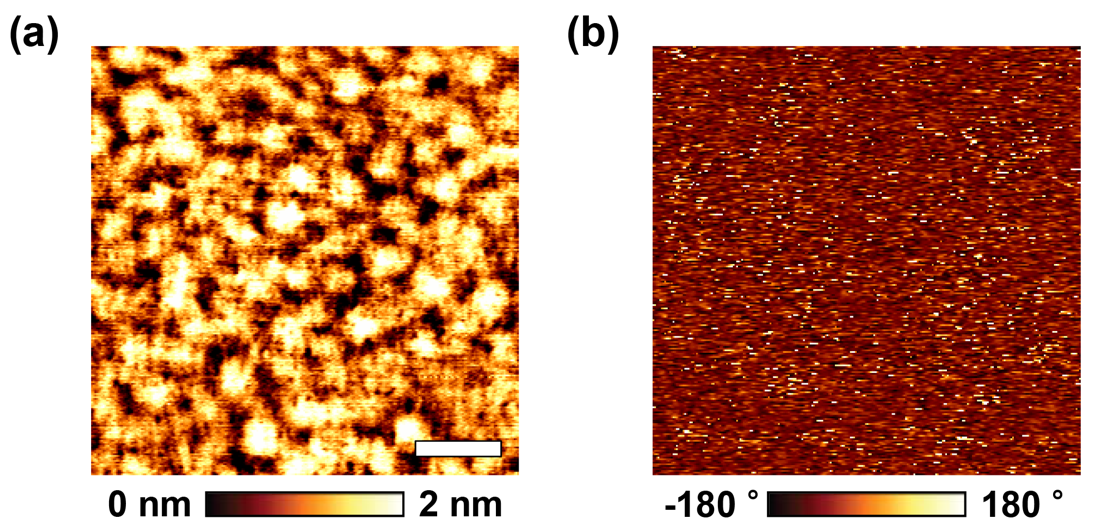


Figure S1. (a) Topography and (b) PFM phase images of the PZT thin films. Scale bar is 400 nm.

The topography of the PZT thin films indicates that a high quality thin film was fabricated, and the PFM phase image shows the preferred upward polarization.


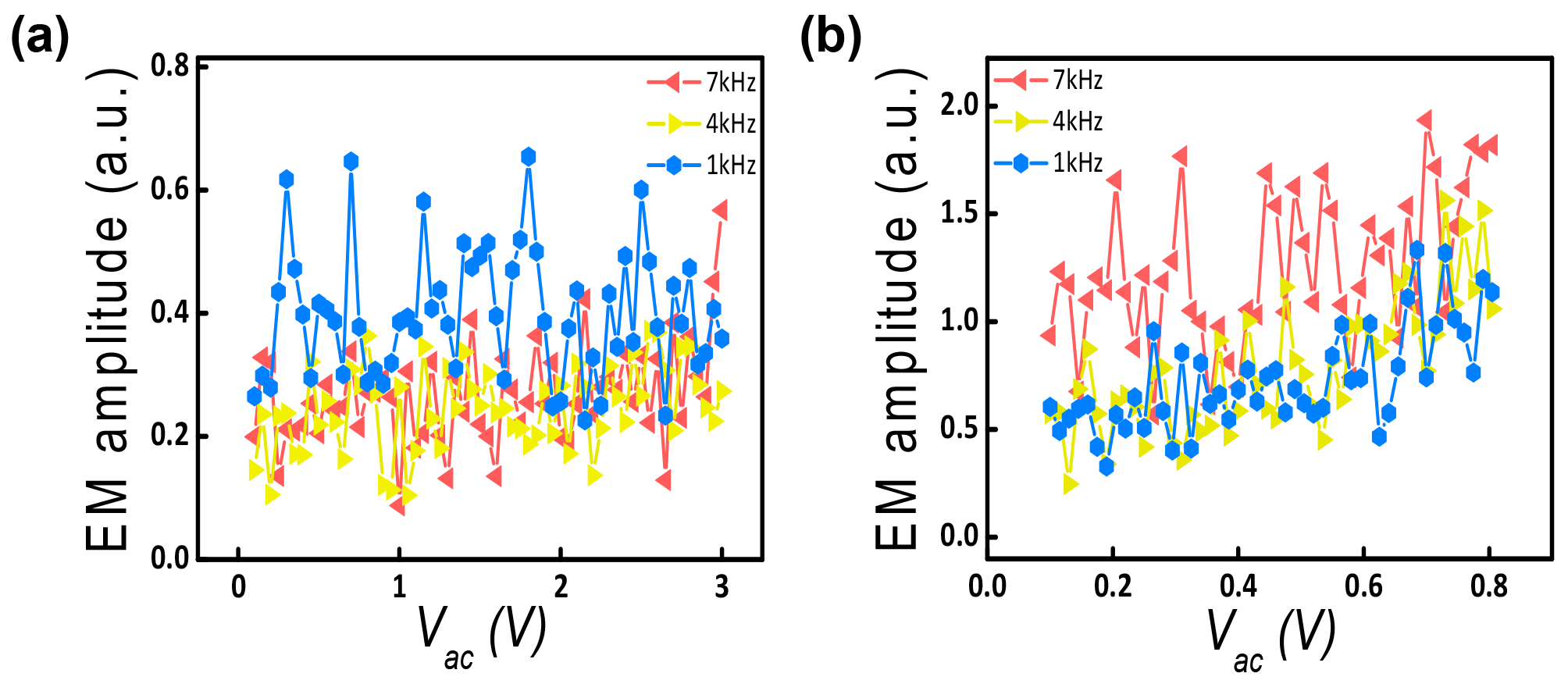


Figure S2. The EM amplitude obtained at the 1, 4 and 7 kHz in (a) the AlPO_4_ phases and (b) PZT thin films.

The EM amplitudes obtained at 1, 4 and 7 kHz in the AlPO_4_ phases and PZT thin films are shown in Fig. S2. In the both cases, linear behavior is not clearly shown. This is because that there is an offset increase, which may be produced by the instrumental noise, in the obtained signal. That is, the initial value of EM amplitude gradually increases according to the decrease of frequency. Since the instrumental offset increase is comparable to observed piezoresponse of the samples, the linear behavior is not clearly shown. In the LAGTPO phases, there is also offset increase with decreasing frequency (see Fig. 3(a)). However, since the increase of EM response is larger than that of instrumental offset, the tendency can be clearly observed in LAGTPO phases. Note that, both the AlPO_4_ phases and the PZT thin film show much clearer behavior at higher than 10 kHz (see Fig. 2).


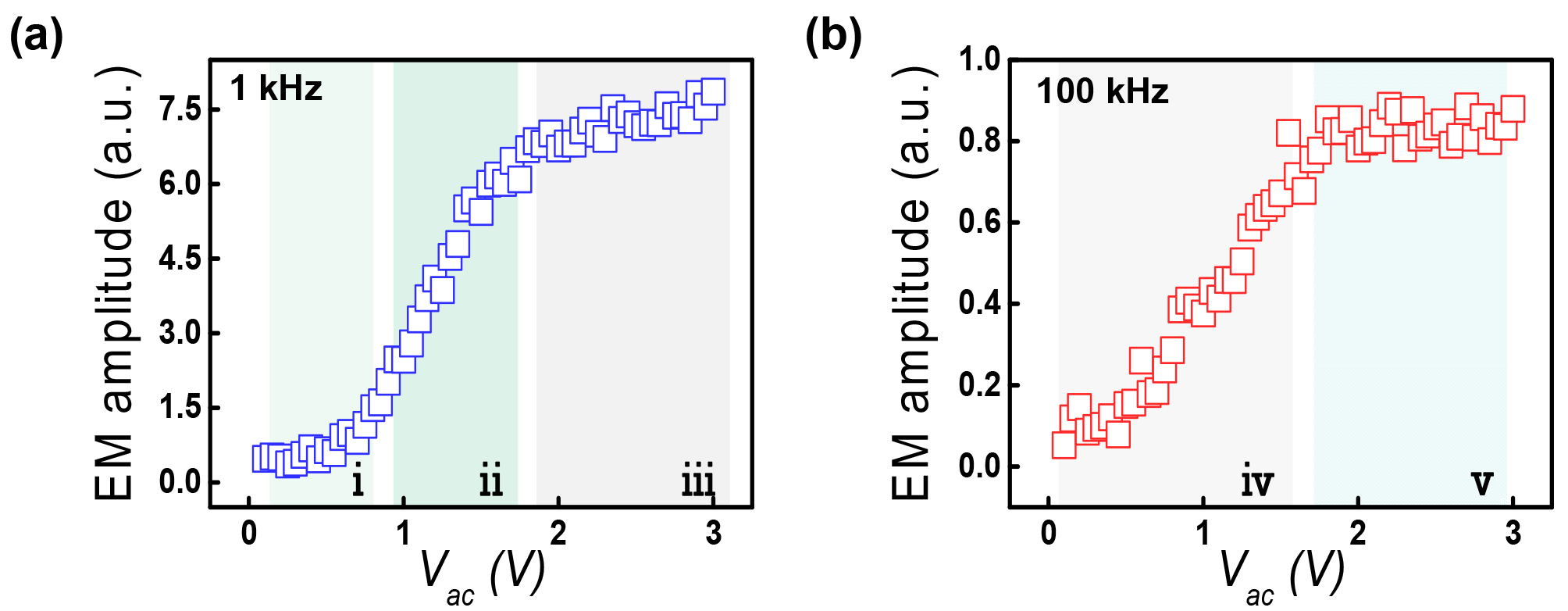


**Figure S3.** The EM amplitude of the LAGTPO phases at two different frequencies: (a) 1 kHz and (b) 100 kHz.

The EM amplitudes obtained for the LAGTPO phases at 1 and 100 kHz show different behavior depending on the frequency, as shown in Fig. S3. The EM amplitude obtained at 1 kHz slightly increases with relatively small ac amplitude, and then it rapidly increases when the ac amplitude becomes larger than a certain value. Finally, it shows a saturation behavior. On the other hand, there is a gradual increase in the EM amplitude at 100 kHz over the range of the ac amplitude until saturation is reached. A detailed explanation on these behaviors is described in Fig. 7.


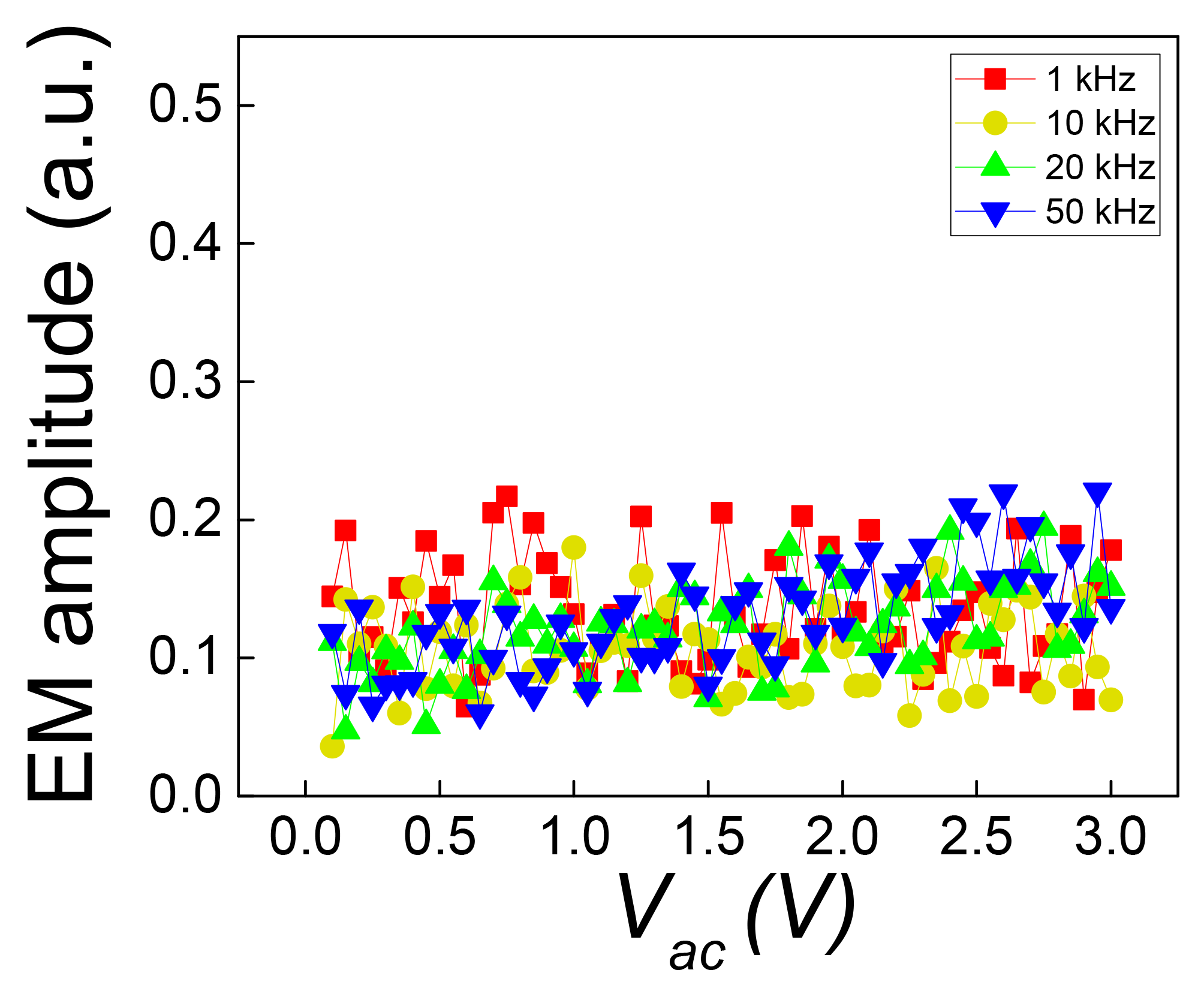


Figure S4. Frequency dependent ac amplitude sweep under the free cantilever state

In order to investigate noise level of the frequency dependence, the frequency dependent ac amplitude sweep under the free cantilever state is presented in Fig. S4. It only shows noisy response. That is, there is no visible dependence on the frequency as well as voltage. Thus, it is concluded that EM amplitude signal of the ac amplitude sweep stems from the response of the sample surface.

**II**. **Flexoelectric, electrostriction and Vegard contributions to the local strains in PFM**

**II.1. Analytical estimates for the 1D-case**

There are three basic frequency dependent contributions to the PFM response, namely flexoelectric, electrostriction and Vegard couplings. The local strain is proportional to

$u_{ij}=\left(s_{ijkl}\sigma_{kl}+F_{ijkl}\frac{\partial P_{k}}{\partial x_{l}}+Q_{ijkl}P_{k}P_{l}+W_{ij}^{a}\left(N_{a}^{-}-N_{a0}^{-}\right)+W_{ij}^{d}\left(N_{d}^{+}-N_{d0}^{+}\right)\right)$ (A.1)

where *s_ijkl_*, $\sigma_{kl}$, $F_{ijkl}$, $Q_{ijkl}$, and $P_{k}\left(\mathbf{r}\right)$ are respectively elastic compliances tensor, elastic stress, flexoelectric stress tensor, electrostriction stress tensor, and polarization component. $W_{ij}^{a}$ and $W_{ij}^{d}$ are the Vegard tensors for acceptors and donors and, correspondingly, $N_{d}^{+}\left(\mathbf{r}\right)$ and $N_{a}^{-}\left(\mathbf{r}\right)$ are the inhomogeneous concentration of ionized donors and acceptors, and $N_{d0}^{+}$ and $N_{a0}^{\text{-}}$ are their equilibrium concentrations.

For the semiconductor film with mixed ionic-electronic conductivity, the electric potential *ϕ* can be found self-consistently from the Poisson equation with the short-circuited electric boundary conditions:

$\left\{\begin{matrix} \varepsilon_{0}\varepsilon_{33}^{b}\frac{\partial^{2}\phi}{\partial z^{2}}=\frac{\partial P_{3}}{\partial z}-\rho(\phi), \\ \phi\left(0\right)=V_{ac}f(t)\sin\left(\omega t\right), \phi\left(h\right)=0. \end{matrix}\right.,$ (A.2a)

Here we regard that the film is placed in a planar capacitor under the voltage $V_{ac}f(t)\sin\left(\omega t\right)$. In order to calculate electrostrictive contribution, one should know the polarization components $P_{m}$ dependence on the electric field $E_{m}$. Thus, we assumed as follows:

(a) A linear dielectric (LD) approximation with the linear relation $P_{m}=\varepsilon_{0}\left(\varepsilon-1\right)E_{m}$ is valid only at small applied voltages.

(b) Alternative Langeven type (LT) approximation is $P_{m}=P_{0}\tanh\left(E_{m}/E_{a}\right)$ where the saturation polarization and acting field are $P_{0}=\varepsilon_{0}\left(\varepsilon-1\right)E_{a}$ and $E_{a}$, respectively. In this case, the polarization amplitude $P_{0}$ and the acting field $E_{a}$ are estimated by assuming a relative dielectric permittivity *ε* of 5 - 50, screening radius *R_d_* of 3 nm and applied voltage *U* of 1 V. Then, the polarization amplitude $P_{0}$ and the acting field $E_{a}$ are approximately obtained as 0.013 - 0.13 C/m^2^ (from $P_{0}=\varepsilon_{0}\left(\varepsilon-1\right)E_{a}$) and ~ 0.3 V/nm (from $E_{a}\sim U/R_{d}$), respectively.

Here we consider that the frequencies $\omega$ are much smaller than the optical frequency and *dc* voltages are much higher than *ac* ones, . Under the assumptions,

$\phi\left(z\right)=\phi_{dc}+\phi_{ac}f(t)\sin\left(\omega t\right)$, $E_{3}\left(z\right)=E_{dc}+E_{ac}f(t)\sin\left(\omega t\right)$, (A.2b)

$\rho\left(\phi\right)=\rho_{dc}+\rho_{ac}f(t)\sin\left(\omega t\right)$ (A.2c)

Below, we will use the depletion/accumulation layer approximation to determine the *dc*components, Debye (or Tomas-Fermi) approximation and *ac* components, in which we are mainly interested in. Each can be written as:

$\rho_{ac}\approx\frac{\phi_{ac}}{h_{d}^{2}}$, $\phi_{ac}=-V_{ac}\frac{\sinh\left(\left(z-h\right)/h_{d}\right)}{\sinh\left(h/h_{d}\right)}$, $E_{3}^{ac}=\frac{V_{ac}}{h_{d}}\frac{\cosh\left(\left(z-h\right)/h_{d}\right)}{\sinh\left(h/h_{d}\right)}$ (A.3)

The semiconductor potential and space charge are distributed in the layer as $0<z<h$and zero outside and the Debye screening length is $h_{d}$. Note that the expression (A.3)contains a continuous transition to the dielectric limitation, $E_{ac}=V_{ac}/h$, at $h_{d}\to\infty$ which occurs at $\omega>>1/\tau_{M}$. A small $h_{d}$, *i.e.* $h_{d}<<h$, corresponds to the electronic instant response to the *ac* field.

Now, we can estimate the film strain and surface displacement that are caused by the flexoelectric coupling in the LD approximation and for LT approximation. The displacement caused by the flexoelectric coupling is given by the integral:

$u_{3}^{F}\left(t\right)=F_{33}\int_{0}^{h} dz\frac{\partial P_{3}}{\partial z}=F_{33}\left(P_{3}(h,t)-P_{3}(0,t)\right)$ (A.4a)

Integration in Eq. (A.4a) gives

$u_{3}^{F}\left(t\right)=\left\{\begin{matrix} \varepsilon_{0}F_{33}\left(\varepsilon-1\right)\frac{V_{ac}}{h_{d}}\left(\frac{1-\cosh\left(h/h_{d}\right)}{\sinh\left(h/h_{d}\right)}\right)f(t)\sin\left(\omega t\right), LD \\ F_{33}P_{0}\tanh\left(\frac{V_{ac}}{E_{a}h_{d}}\left(\frac{1-\cosh\left(h/h_{d}\right)}{\sinh\left(h/h_{d}\right)}\right)f(t)\sin\left(\omega t\right)\right), LT \end{matrix}\right.$ (A.4b)

As one can see from Eq. (A.4b), the flexoelectric coupling contribution is proportional to *ac* voltage. The response depends linearly on the applied ac voltage at small voltages and then saturates for the LT case. Thus, this case can be distinguished as two different regimes (linear and sublinear saturation) at low frequencies. In addition, the *ac* contribution tends to become zero in dielectric limit, $h_{d}\to\infty$. Therefore, the flexoelectric contribution induced by the *ac* component of the PFM response can be neglected in the dielectric limit. The displacement caused by the electrostriction coupling is given by the integral

$u_{3}^{Q}\left(t\right)=Q_{33}\int_{0}^{h} P_{3}^{2}dz$ (A.5)

The electrostrictive contribution in the LD approximation is written as

$u_{3}^{Q}\left(t\right)=Q_{33}\left(\varepsilon_{0}\left(\varepsilon-1\right)\right)^{2}\int_{0}^{h} \left(E_{3}^{ac}f(t)\sin\left(\omega t\right)\right)^{2}dz\approx u_{3}^{0}f^{2}(t)\frac{1-\cos\left(2\omega t\right)}{2}$ (A.6a)

where

$u_{3}^{0}=-Q_{33}\left(\varepsilon_{0}\left(\varepsilon-1\right)\right)^{2}\left(\frac{V_{ac}}{2h_{d}}\right)^{2}\frac{2h+h_{d}\sinh\left(2h/h_{d}\right)}{2\sinh^{2}\left(h/h_{d}\right)}\to_{h_{d}\to\infty} -\frac{Q_{33}}{2}\left(\varepsilon_{0}\left(\varepsilon-1\right)\right)^{2}\frac{V_{ac}^{2}}{h}$ (A.6b)

The electrostrictive contribution in the LT approximation can be calculated as:

$u_{3}^{0}=-Q_{33}P_{0}^{2}\int_{0}^{h} \tanh^{2}\left(\frac{V_{ac}}{E_{a}h_{d}}\frac{\cosh\left(\left(z-h\right)/h_{d}\right)}{\sinh\left(h/h_{d}\right)}\right)dz\to_{h_{d}\to\infty} -\frac{Q_{33}}{2}\left(\varepsilon_{0}\left(\varepsilon-1\right)\right)^{2}E_{a}^{2}h\tanh^{2}\left(\frac{V_{ac}}{E_{a}h}\right)$ (A.6c)

The contribution depends quadratically on the applied ac voltage at small voltages, then depends linearly and then saturates for the LT case. Hence, this case can be distinguished for three regimes (parabolic, quasi-linear and sublinear saturation) at low frequencies.

The displacement caused by the Vegard contribution is given by the integral:

$u_{3}^{V}\left(t\right)=\int_{0}^{h} dz\left(W_{33}^{a}\left(N_{a}^{-}-N_{a0}^{-}\right)+W_{33}^{d}\left(N_{d}^{+}-N_{d0}^{+}\right)\right)$ (A.7)

The contribution seems to proportional with the total charge of each species,^1^ which is identically zero for the case when the top and substrate electrodes are ionically blocking, because the identities $\int_{0}^{h} dz\left(N_{a}^{-}-N_{a0}^{-}\right)=0$ and $\int_{0}^{h} dz\left(N_{d}^{+}-N_{d0}^{+}\right)=0$ are valid for the blocking electrodes. Note that it is not true the case for the 2D problem.

# II. 2. Numerical results for the 3D case corresponding to PFM geometry

To gain insight into the mechanisms of the local EM amplitude, *i.e.*, PFM image formation, we consider a cylindrical problem of nonlinear drift-diffusion kinetics that allow for the Vegard, electrostriction, steric limit for mobile ions concentration and Fermi-Dirac distribution function for electron density, thus including the most common form of charge species that are nonlinearly inherent to the system. The finite element method performed for the cylindrical geometry at different frequencies and bias voltage amplitudes provides the concentration and strain distribution registered by the PFM ^3^.

The typical geometry of PFM with an axially symmetric tip is shown in Fig. 7(a). All of the physical quantities depend only on the distances *z* from the tip-surface interface and the polar radius *r* (2D problem). The mobile positively charged defects, such as oxygen vacancies or cations, and free electrons are inherent to the film. The redistribution of the mobile charge carriers creates an internal electric field of which the radial and normal components, $E_{r}=-\partial\phi/\partial r$ and $E_{z}=-\partial\phi/\partial z$, are determined from the Poisson equation for electric potential *ϕ*, written in the cylindrical coordinate frame as:

$\varepsilon_{0}\varepsilon\left(\frac{\partial^{2}\phi}{\partial r^{2}}+\frac{1}{r}\frac{\partial\phi}{\partial r}+\frac{\partial^{2}\phi}{\partial z^{2}}\right)=-e\left(Z_{d}N_{d}^{+}\left(\phi\right)-n\left(\phi\right)\right)$ (B.1)

where *ε_o_*, *ε*, *e, n,* $N_{d}^{+}$, and $Z_{d}$ are the dielectric permittivity of vacuum, dielectric permittivity of mixed ionic-electronic conductor (MIEC), the electron charge, electron density, positively charged defect concentration and their charge in the units of electron charge, respectively. For the particular case of the electrode film of thickness *h,* the electric potential satisfies fixed boundary conditions at the electrodes, $\left.\phi\right|_{z=0}=0$, $\left.\phi\right|_{z=h}=U(r,t)$, and vanishes at infinity, $\left.\phi\right|_{r\to\infty}=0$. We use the Gaussian form of the periodic voltage *U* applied to the tip electrode, $U(r,t)=U_{0}\exp\left(-r^{2}/r_{0}^{2}\right)\sin\left(\omega t\right)$, and regard that the tip lateral size *r_o_* is much smaller than the size of the computation cell. Background for the approximation is given in reference 4.

The continuity equation for a mobile charged defect concentration $N_{d}^{+}$ is written as:

$\frac{\partial N_{d}^{+} }{\partial t}+ \frac{1}{eZ_{d}}\left(\frac{1}{r}\frac{\partial(rJ_{r}^{d}) }{\partial r}+\frac{\partial J_{z}^{d} }{\partial z}\right)=G_{d}\left(N_{d}^{+},n\right)$ (B.2)

The defect current radial and normal components are proportional to the gradients of the electrochemical potentials level $\zeta_{d}$, namely $J_{r}^{d}=-eZ_{d}\eta_{d}N_{d}^{+} \left(\partial\zeta_{d}/\partial r\right)$ and $J_{z}^{d}=-eZ_{d}\eta_{d}N_{d}^{+} \left(\partial\zeta_{d}/\partial z\right)$, where $\eta_{d}$ is the mobility coefficient that is regarded as a constant. The boundary conditions for the defect are blocking $\left.J_{d}\right|_{z=0}=0$; $\left.J_{d}\right|_{z=h}=0$;$\left.J_{d}\right|_{r=R}=0$. The function $G_{d}\left(N_{d}^{+},n\right)\propto-\gamma_{d}N_{d}^{+}n+\gamma_{T}\left(N_{d}^{0}-N_{d}^{+}\right)$ describes the electron trapping due to ionized defects and their generation due to non-ionized ones. The function is primarily neglected in numerical simulations because its concrete form and the values of the trapping coefficients are typically unknown. The electrochemical potential level $\zeta_{d}$ is defined as:

$\zeta_{d}=-E_{d}-W_{ij}^{d}\sigma_{ij}+eZ_{d}\phi+k_{B}T\ln\left(\frac{N_{d}^{+}}{N_{d}^{S}-N_{d}^{+}}\right)$. (B.3)

where $E_{d}$, $\sigma_{ij}$ *, T, k_B_* and $W_{ij}^{d}$ are the impurity level, elastic stress tensor, absolute temperature, Boltzmann constant and Vegard strain tensor, respectively. For the case with a diagonal Vegard strain tensor, we have $W_{ij}^{d}\sigma_{ij}\equiv W\sigma$, where $\sigma=\sigma_{zz}+\sigma_{rr}+\sigma_{\phi\phi}$. The absolute values of *W* for the ABO_3_ compounds can be estimated from reference values^1,2^ as $\left|W\right|$ (1 − 50) Å^3^. The maximal stoichiometric concentration of the defects ($N_{d}^{S}$) in Eq.(B.3) takes into account the steric effects.

The continuity equation for the electron current is written as:

$\frac{\partial n}{\partial t}-\frac{1}{e}\left(\frac{1}{r}\frac{\partial(rJ_{r}^{e}) }{\partial r}+\frac{\partial J_{z}^{e} }{\partial z}\right)=G_{e}\left(N_{d}^{+},n\right)$ (B.4)

The electron current components are $J_{r}^{e}=e\eta_{e}n \left(\partial\zeta_{e}/\partial r\right)$ and $J_{z}^{e}=e\eta_{e}n \left(\partial\zeta_{e}/\partial z\right)$, where $\eta_{e}$ and $\zeta_{e}$ are the electron mobility coefficient and the electro-chemical potential, respectively. The function $G_{e}\left(N_{d}^{+},n\right)=G_{d}\left(N_{d}^{+},n\right)-n/\tau_{n}$ describes the electron trapping by the ionized defect and includes its finite lifetime. For the reasons mentioned above, the function will be neglected in numerical simulations. The boundary conditions for the electrons are taken in a linearized Chang-Jaffe form,^1^ $\left.\left(J_{z}^{e}-\xi_{0}\left(n-n_{b}\right)\right)\right|_{z=0}=0$, $\left.\left(J_{z}^{e}+\xi_{h}\left(n-n_{b}\right)\right)\right|_{z=h}=0$, $\left.J_{e}\right|_{r\to\infty}=0$, where $\xi_{0}$ and $\xi_{h}$ are positive rate constants related to the surface recombination velocity. The continuous approximation for the electron density in the conduction band is consistent with the following expression for electro-chemical potential $\zeta_{e}$:

$\zeta_{e}\approx E_{C}-\Xi_{ij}\sigma_{ij}+k_{B}T F_{1/2}^{-1}\left(\frac{n\left(\phi\right)}{N_{C}}\right)-e\phi$ (B.5)

where $E_{F}$, $E_{C}$, $\Xi_{ij}$, $F_{1/2}^{-1}$, and $m_{n}$ are the Fermi energy level, the bottom of the conductive band, a deformation potential tensor that is also regarded to be diagonal for the numerical estimation, the function inverse to the Fermi integral $F_{1/2}\left(\xi\right)=\frac{2}{\sqrt{\pi}}\int_{0}^{\infty} \frac{\sqrt{\zeta}d\zeta}{1+\exp\left(\zeta-\xi\right)}$; effective density of states in the conductive band , where $m_{n}$ is effective mass of electron, respectively. The electro-chemical potential $\zeta_{e}$ tends to be close to the Fermi energy level $E_{F}$ in equilibrium. The expression provides the thermodynamic distribution of electron density under the condition $\zeta_{e}=-E_{F}$.

The frequency dependent contribution in the EM amplitude can be calculated as follows. We used the generalized Hooke’s law for a chemically active elastic solid media relating the concentration deviation from the average with mechanical stress tensor σ*_ij_* and elastic strain *u_ij_* where we suppose that only two kinds of species contribute to the elastic field and denote $W_{ij}\equiv W_{ij}^{d}$ and $\Xi_{ij}\equiv\Xi_{ij}^{C}$. Since the typical intrinsic resonance frequencies of material are in the GHz range, well above the practically important limits both in terms of the ion dynamics and the PFM-based detection of localized mechanical vibrations, one can use a static equation of mechanical equilibrium, $\partial\sigma_{ij}/\partial x_{j}=0$, to model the mechanical phenomena (here, we have neglected the first and second time derivatives). This leads to an equation for the mechanical displacement vector *u_i_* in the bulk of the film:

$c_{ijkl}\left(\frac{\partial^{2}u_{k}}{\partial x_{j}\partial x_{l}}-W_{kl} \frac{\partial\delta N_{d}^{+}}{\partial x_{j}}-\Xi_{kl} \frac{\partial\delta n}{\partial x_{j}}-F_{klmn}\frac{\partial^{2}P_{m}}{\partial x_{j}\partial x_{n}}-Q_{iklmn}\frac{\partial\left(P_{m}P_{n}\right)}{\partial x_{j}}\right)=0$ (B.6)

Here, we have introduced $\delta N_{d}^{+}\left(\mathbf{r},t\right)=N_{d}^{+}\left(\mathbf{r},t\right)-N_{d0}^{+}$ and $\delta n\left(\mathbf{r},t\right)=n\left(\mathbf{r},t\right)-n_{e}$. As mentioned above, the polarization amplitude $P_{0}$ and the acting field $E_{a}$, approximated as 0.013–0.13 C/m^2^ (from $P_{0}=\varepsilon_{0}\left(\varepsilon-1\right)E_{a}$) and ~ 0.3 V/nm (from $E_{a}\sim U/R_{d}$), are used for the calculation. The boundary condition on the free surface *z* = 0 of the film is $\sigma_{3j}\left(z=0,t\right)=0$. The surface *z*=*h* is clamped to a rigid substrate, thus the boundary condition of *z=h* is $\left.u_{k}\right|_{z=h}=0$.

**Table 1.**

| **Parameter or ratio** | **Numerical value** | **Comment** |
| --- | --- | --- |
| *h* (nm) | ~40 | Sample thickness |
| *r*_0_ (nm) | 30 - 150 | Probe radius |
| *E_a_* (V/m) | 5.4×10^10^ | Threshold field |
| $t_{d}/t_{e}\equiv\eta_{e}/\eta_{d}$ | 10 | Donor-to-electron relaxation times ratio |
| ω (Hz) | 1 - 1000 | Angular frequency |
| $E_{C}$ (eV) | 0 | Conduction band position |
| $E_{d}$ (eV) | −0.1 | Donor level |
| $E_{F}$ (eV) | N/A | Fermi energy is determined self-consistently from the electroneutrality condition |
| $m_{n}/m_{0}$ | 0.5 | Effective –to-free electron mass ratio |
| *W* (Å^3^) | 10 | Vegard coefficient |
| $s_{11}+s_{12}$ (Pa^−1^) | 3.44×10^−12^ | Elastic compliances |
| $N_{d}^{0}$ (m^−3^) | 10^24^ | Donors maximal concentration |
| *T* (K) | 298 | Room temperature |
| $\varepsilon$ | 5 | Dielectric permittivity |
| $D_{i}$ (cm^2^/s) | 10^−14^ | Ions diffusion coefficient |
| $D_{e}$ (cm^2^/s) | 10^−12^ | Electrons diffusion coefficient |
| $\eta_{e}$ | 2.43×10^8^ | Electrons mobility |
| $\eta_{i}$ | 2.43×10^6^ | Donors mobility |
| $N_{C}$ | 4.39×10^24^ | Effective density of states in the conductive band |
| $Y$ | 0.25 | Poisson’s ration |
| $\chi_{11}$ | 50 | Susceptibility |
| $\chi_{22}$ | 50 | Susceptibility |
| $Q_{11}$ (m^4^/C^2^) | 0.081 | Electrostriction tensor |
| $Q_{12}$ ( m^4^/C^2^) | -0.0295 | Electrostriction tensor |
| $Q_{44}$ ( m^4^/C^2^) | 0.0675 | Electrostriction tensor |
| $F_{11}$ ( m^3^/C) | $15\cdot10^{-12}$ | Flexoelectric tensor |
| $F_{22}$ (m^3^/C) | $9\cdot10^{-12}$ | Flexoelectric tensor |
| $F_{44}$ (m^3^/C) | $6\cdot10^{-12}$ | Flexoelectric tensor |

**References**

1. Morozovska A. N. et al. Thermodynamics of electromechanically coupled mixed ionic-electronic conductors: Deformation potential, Vegard strains, and flexoelectric effect. *Phys. Rev. B* **83**, 195313 (2011).

2. Freedman D. A., Roundy D. & Arias T. A. Elastic effects of vacancies in strontium titanate: Short- and long-range strain fields, elastic dipole tensors, and chemical strain. *Phys. Rev. B* **80**, 064108 (2009)

3. Chang H. -C. & Jaffe G. J. Polarization in Electrolytic Solutions. Part I. Theory. *J. Chem. Phys.* **20**, 1071 (1952).

4. Varenyk O.V. et al. Self-consistent modelling of electrochemical strain microscopy in mixed ionic-electronic conductors: Nonlinear and dynamic regimes. *J. Appl. Phys.* **118**, 072015 (2015).
